# Interleukin-8 -251A/T gene polymorphism and lung cancer susceptibility: a meta-analysis

**DOI:** 10.1111/jcmm.12466

**Published:** 2015-03-27

**Authors:** Xiao-Bin Wang, Yun-Song Li, Jie Li, Yi Han, Zhi-Dong Liu

**Affiliations:** a Department of Thoracic Cardiovascular Surgery, Inner Mongolia Forestry General HospitalYakeshi, China; b Department of Thoracic Surgery, Beijing Chest Hospital, Capital Medical UniversityBeijing, China; c Department of Oncology, Beijing Chest Hospital, Capital Medical UniversityBeijing, China

**Keywords:** Interleukin-8, polymorphism, lung cancer, susceptibility, meta-analysis

## Abstract

Many studies have examined the association between the *interleukin-8 -251T/A* (rs4073) gene polymorphism and lung cancer risk in various populations, but the results have been inconsistent. In this meta-analysis, PubMed was searched for case–control studies published through 01 December 2013. The data were extracted, and pooled odds ratios (OR) with 95% confidence intervals (CI) were calculated. We assessed six published studies on the association between the *interleukin-8 -251T/A* polymorphism and lung cancer risk. The included studies yielded a total of 3265 lung cancer cases and 3607 controls. For the homozygous A/A and A allele carriers (T/A + A/A), the pooled ORs for all studies combining 3265 cases and 3607 controls were 1.03 (95% CI = 0.92–1.14; *P* = 0.235 for heterogeneity) and 1.07 (95% CI = 0.96–1.19; *P* = 0.245 for heterogeneity) when compared with the homozygous wild-type genotype (T/T). When the analysis was stratified by ethnicity, significant risks were found among Asians for both the A allele carriers and the homozygous A/A individuals. However, no significant associations were found in non-Asian populations using any of the genetic models. This meta-analysis suggests that the *interleukin-8 -251A* allele confer an increased risk for the development of lung cancer among Asians.

## Introduction

Lung cancer remains the deadliest cancer worldwide despite improvements in diagnostic and therapeutic techniques 1. The incidence of lung cancer has yet to peak in many parts of the world, particularly in China, where it has become a major public health challenge 2. The mechanism of lung carcinogenesis is still not fully understood. In addition to smoking status, which has been established as the most important single factor in causing lung cancer, host factors, including genetic polymorphisms, have received increasing attention in studies on the tumourigenesis of lung cancer 3. Many environmental carcinogens require metabolic activation by various drug-metabolizing enzymes.

*Interleukin-8* (IL-8), a member of the chemokine family, is a chemoattractant of neutrophils and lymphocytes 4,5. IL-8 is produced by a wide range of normal cells including monocytes, neutrophils, fibroblasts and endothelial cells 6. Initially characterized for its leucocyte chemotactic activity, IL-8 is mainly involved in the initiation and amplification of acute inflammatory reactions 7. In addition, IL-8 is produced by several types of tumour cells 8 and has been shown to be involved in angiogenesis and neovascularization-dependent tumour growth 9,10. IL-8 is also overexpressed in a variety of human tumours and is involved in tumour invasion and metastasis 11–13. The predominant homozygous genotype, the heterozygous genotype and the rare homozygous genotype of the IL-8 -251T/A (rs4073) polymorphism are designated as T/T, A/T and A/A, respectively.

Many studies have investigated the associations between the IL-8 -251T/A (rs4073) gene polymorphism and lung cancer risk, but the findings have been inconsistent. A single study may not have sufficient power to detect a small effect of the polymorphism on lung cancer, particularly when the sample size is relatively small. Different types of study populations may also contribute the disparate findings. Hence, we performed a meta-analysis of all eligible studies to derive a more precise estimation of the associations of the IL-8 -251T/A (rs4073) polymorphism with lung cancer.

## Materials and methods

### Publication search

The electronic database PubMed was searched for studies to include in the present meta-analysis using the following terms: ‘IL-8’ or ‘interleukin-8’, ‘-251T/A’ or ‘rs4073’, ‘polymorphism’ and ‘lung cancer’. An upper date limit of 01 December 2013 was applied; no lower date limit was used. The search was performed without any restrictions on language and was focused on studies that had been conducted in humans. Concurrently, the reference lists of the reviews and retrieved articles were searched manually. Only full-text articles were included. When the same patient population appeared in several publications, only the most recent or complete study was included in this meta-analysis.

### Inclusion criteria

The included studies had to meet the following criteria: (*i*) evaluated the IL-8 - 251T/A polymorphism and lung cancer risk; (*ii*) case–control studies and (*iii*) supplied the number of individual genotypes for IL-8 -251T/A in lung cancer cases and controls.

### Data extraction

Information was carefully extracted from all eligible publications by two authors independently according to the inclusion criteria listed above. Disagreement was resolved by discussion between the two authors.

The following data were collected from each study: first author's surname, year of publication, ethnicity, total numbers of cases and controls, and numbers of cases and controls with the TT, TA and AA genotypes. If data from any of the above categories were not reported in the primary study, the items were treated as ‘not applicable’. We did not contact the author of the primary study to request the information. Different ethnicities were categorized as Asian and non-Asian. We did not require a minimum number of patients for a study to be included in our meta-analysis.

### Statistical analysis

Odds ratios (ORs) with 95% CI were used to assess the strength of the association between the IL-8 -251T/A polymorphism and lung cancer risk. The pooled ORs for the risk associated with the genotypes A/A and A allele carriers (A/T + A/A) with the T/T genotype were calculated. Subgroup analyses were performed based on ethnicity. The assumption of heterogeneity was checked by the chi-squared based *Q*-test 14. A *P*-value greater than 0.10 for the *Q*-test indicates a lack of heterogeneity among the studies, so the pooled OR estimate of each study was calculated by the fixed-effects model (the Mantel–Haenszel method) 15. Otherwise, the random-effects model (the DerSimonian and Laird method) was used 16. One-way sensitivity analyses were performed to assess the stability of the results; namely, a single study in the meta-analysis was deleted each time to reflect the influence of the individual data set on the pooled OR 17. An estimate of potential publication bias was carried out with a funnel plot, in which the standard error of the log (OR) of each study was plotted against its log (OR). An asymmetric plot suggests a possible publication bias. The funnel plot asymmetry was assessed using Egger's linear regression test, a linear regression approach to measure the funnel plot asymmetry on the natural logarithm scale of the OR. The significance of the intercept was determined by the *t*-test suggested by Egger (*P* < 0.05 was considered representative of statistically significant publication bias) 18. All of the calculations were performed with STATA version 11.0 (Stata Corporation, College Station, TX, USA).

## Results

### Study characteristics

A total of six publications involving 3265 lung cancer cases and 3607 controls met the inclusion criteria and were included in the analysis 19–24. Table1 presents the main characteristics of these studies. Among the six publications, all were published in English. The sample sizes ranged from 231 to 4260. Almost all of the cases were histologically confirmed. The controls were mainly healthy populations. There were two groups of Asians and four groups of non-Asian population.

**Table 1:** Main characteristics of studies investigated the association between IL-8 -251 T/A polymorphisms and lung cancer risk in the meta-analysis

First author-year	Ethnicity (country of origin)	Study design	Sample size (case/control)	Lung cancer case	Controls
				TT/TA/AA	TT/TA/AA
Bhat 2013	Asian (India)	HCC	190/200	37/67/86	55/66/79
Rafrafi 2013	Tunisia	PCC	170/225	80/100/45	86/65/19
Lee 2007	Asian (China)	PCC	119/112	42/55/22	48/49/15
Vogel 2008	Denmark	PCC	403/744	91/203/109	161/364/219
Campa 2005	France	HCC	2144/2116	578/1081/485	574/1084/458
Campa 2005	Norway	HCC	239/210	71/119/49	54/112/44

HCC, hospital-based case–control studies; PCC, population-based case–control studies.

### Meta-analysis results

**Figure 1:**
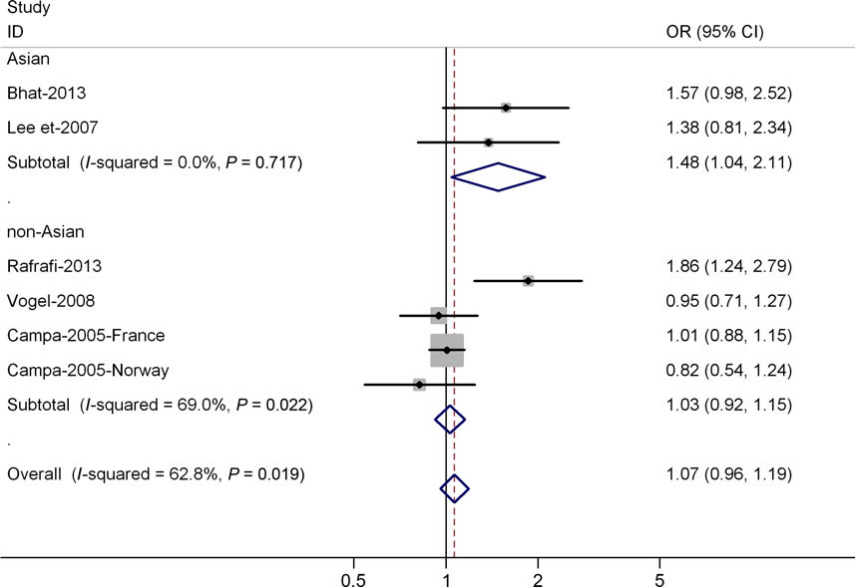
Forest plot (fixed-effects model) of lung cancer risk associated with IL-8 -251 T/A polymorphism for (TA + AA) *versus*TT. Each box represents the OR point estimate, and its area is proportional to the weight of the study. The diamond (and broken line) represents the overall summary estimate, with CI represented by its width. The unbroken vertical line is set at the null value (OR = 1.0).

Table2 lists the main results of this meta-analysis. Overall, for the homozygous A/A and A allele carriers (T/A + A/A), the pooled ORs for all studies, combining 3265 cases and 3607 controls, were 1.03 (95% CI = 0.92–1.14; *P* = 0.235 for heterogeneity) and 1.07 (95% CI = 0.96–1.19; *P* = 0.245 for heterogeneity) (Fig.1) when compared with the homozygous wild-type genotype (T/T). When the analysis was stratified by ethnicity, significant risk was found among Asian populations for both the A allele carriers (OR = 1.48, 95% CI = 1.04–2.11; *P* = 0.030 for heterogeneity) and homozygous A/A individuals (OR = 1.35; 95% CI = 1.02–1.92; *P* = 0.090 for heterogeneity). Among non-Asian populations, no significant association was found for A/A *versus* T/T (OR = 1.12; 95% CI = 0.72–1.25; *P* = 0.284 for heterogeneity) or for A allele carriers *versus* T/T (OR = 1.03; 95% CI = 0.92–1.15; *P* = 0.602 for heterogeneity).

**Table 2:** Main results of pooled odds ratios (OR) with confidence interval (CI) in the meta-analysis

	Number of cases/controls	(TA + AA) *versus* TT			AA *versus* TT		
		OR (95% CI)	*P*	*P* (*Q*-test)	OR (95% CI)	*P*	*P* (*Q*-test)
Total	3265/3607	1.07 (0.96–1.19)	0.019	0.245	1.03 (0.92–1.14)	0.027	0.235
Asian	309/312	1.48 (1.04–2.11)	0.717	0.030	1.35 (1.02–1.92)	0.355	0.090
Non-Asian	2956/3295	1.03 (0.92–1.15)	0.022	0.602	1.12 (0.72–1.25)	0.003	0.284

*P*(*Q*-test), *P*-value of *Q*-test for heterogeneity test; OR, odds ratio; CI, confidence interval.

### Sensitivity analyses

A single study involved in the meta-analysis was deleted each time to reflect the influence of the individual data set on the pooled ORs, and the corresponding pooled ORs were not significantly altered (data not shown).

### Publication bias

**Figure 2:**
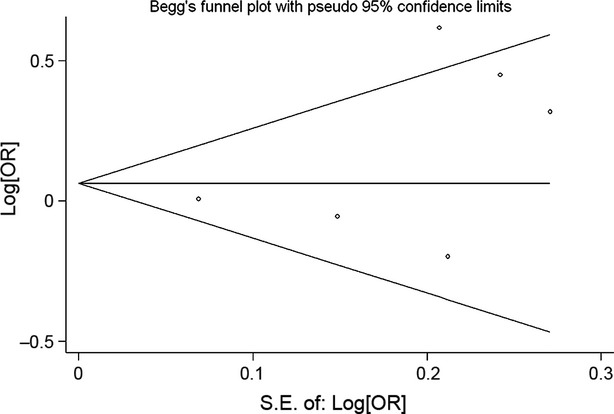
Begg's funnel plot of IL-8 -251 T/A polymorphism and lung cancer risk for (TA + AA) *versus*TT.

Begg's funnel plot and Egger's test were performed to access the publication bias of the literature. The evaluation of publication bias for TA + AA *versus* TT using Egger's test demonstrated that the publication bias was not significant (*P* = 0.283). In addition, the funnel plots for publication bias (Fig.2) did not show asymmetry. These results did not indicate a potential for publication bias.

## Discussion

The IL-8 gene is located on chromosome 4q13-21 and consists of four exons, three introns and a proximal promoter region. The coding sequence of this gene exhibits several functional polymorphisms, and 15 of these have been characterized 25. It is well recognized that there is a range of individual susceptibility to the same type of cancer, even with identical environmental exposure. Host factors, including polymorphisms of genes involved in carcinogenesis, may have accounted for this difference. Therefore, genetic susceptibility to cancer has been a focus for research in the scientific community. Recently, variants of the IL-8 gene involved in the aetiology of several cancers have drawn increasing attention. Recently, two meta-analysis 26,27 about Interleukin-8 -251A > T polymorphism indicated that the polymorphism interleukin-8 -251A/T is associated with a significantly increased risk of gastric carcinogenesis, breast cancer and nasopharyngeal carcinoma. This meta-analysis summarize all the available data on the association between IL-8 -251T/A polymorphism and lung cancer risk, including a total of 3265 lung cancer cases and 3607 controls. Our results indicated a lack of association between the IL-8 -251T/A polymorphism and lung cancer risk among all populations, however, the risk was significantly higher among the Asian population for all genetic models.

When stratified according to ethnicity, Asian carriers of the A allele and homozygous A/A individuals showed an increased risk of lung cancer compared with those with the T/T genotype. However, among the non-Asian population, no significant associations were found with any of the genetic models. These findings indicate that the IL-8 -251T/A polymorphism may be important in lung cancer patients with a specific ethnicity, and the effect of the A allele on the risk of lung cancer may differ by ethnicity. Population stratification is an area of concern, and it can lead to spurious evidence for the association between a marker and a disease, suggesting possible roles for ethnic differences in genetic background and the environment 28. In addition, it is also likely that the observed ethnic differences may be as a result of chance because studies with a small sample size may have insufficient statistical power to detect a slight effect, or they may result in inconsistent assessments. However, only two Asian studies were included in our meta-analysis. The observed ethnic differences may also be a result of chance because studies with small sample size are likely to be insufficiently powered to detect a slight effect.

The mechanism by which the A allele of IL-8 -251T/A may reduce the lung cancer risk is not clear. The T/A single nucleotide polymorphism was identified in the promoter region of the IL- 8 gene 251 base pairs upstream of the transcriptional start site, and *in vitro* assays have demonstrated that it influences the production of IL-8 and affects the transcriptional activity of the IL-8 promoter 29,30. Almost all of the previous reports have demonstrated that the -251A allele is directly associated with higher IL-8 transcription activity 30,4,31. Thus, the A/A genotypes may have the ability to metabolically activate mutagens and carcinogens.

Some limitations of this meta-analysis should be acknowledged. First, heterogeneity is a potential problem when interpreting the results of a meta-analysis. Although we minimized the likelihood of this issue by performing a careful search for published studies, using explicit criteria for study inclusion and rigorously performing the data extraction and analysis, significant between-study heterogeneity still existed in almost each comparison. The presence of heterogeneity can result from differences in the selection of controls, age distribution, lifestyle factors and so on. Although most of the controls were selected from healthy populations, some studies had selected controls among friends or family of lung cancer patients or patients with other diseases. Second, only published studies were included in this meta-analysis. The presence of publication bias indicates that non-significant or negative findings may be unpublished. Last, our results were based on unadjusted estimates, whereas a more precise analysis should be conducted if individual data were available, which would allow for the adjustment by other covariates including age, ethnicity, family history, environmental factors and lifestyle.

In conclusion, this meta-analysis suggests that the IL-8 -251T/A polymorphism is not associated with lung cancer risk among all population, however the A allele is an increased risk factor for developing lung cancer among Asians. In addition, it is necessary to conduct large blind trials using standardized, unbiased methods, homogeneous lung cancer patients and well-matched controls.

## Conflicts of interest

The authors confirm that there are no conflicts of interest.
